# Plant diversity and structure describe the presence of a new, threatened Australian marsupial within its highly restricted, post-fire habitat

**DOI:** 10.1371/journal.pone.0182319

**Published:** 2017-08-10

**Authors:** Eugene D. Mason, Jennifer Firn, Harry B. Hines, Andrew M. Baker

**Affiliations:** 1 Earth, Environmental and Biological Sciences School, Queensland University of Technology, Brisbane, Queensland, Australia; 2 Queensland Museum, South Brisbane, Qld, Australia; 3 Queensland Parks and Wildlife Service, Department of National Parks, Sport and Racing, Level 19, Brisbane, Queensland, Australia; University of Sydney, AUSTRALIA

## Abstract

Management of critical habitat for threatened species with small ranges requires location-specific, fine-scale survey data. The silver-headed antechinus (*Antechinus argentus*) is known from only two isolated, fire-prone locations. At least one of these populations, at Kroombit Tops National Park in central-eastern Queensland, Australia, possesses a very small range. Here, we present detailed vegetation species diversity and structure data from three sites comprising the known habitat of *A*. *argentus* at Kroombit Tops and relate it to capture data obtained over two years. We found differences in both vegetation and capture data between burnt and unburnt habitat. Leaf litter and grasstrees (*Xanthorrhoea johnsonii*) were the strongest vegetative predictors for *A*. *argentus* capture. The species declined considerably over the two years of the trapping study, and we raise concern for its survival at Kroombit Tops. We suggest that future work should focus on structural vegetative variables (specifically, the diameter and leaf density of grasstree crowns) and relate them to *A*. *argentus* occurrence. We also recommend a survey of invertebrate diversity in grasstrees and leaf litter with a comparison to *A*. *argentus* prey. The data presented here illustrates how critical detailed monitoring is for planning habitat management and fire regimes, and highlights the utility of a high-resolution approach to habitat mapping. While a traditional approach to fire management contends that pyrodiversity encourages biodiversity, the present study demonstrates that some species prefer long-unburnt habitat. Additionally, in predicting the distribution of rare species like *A*. *argentus*, data quality (i.e., spatial resolution) may prevail over data quantity (i.e., number of data).

## Introduction

In Australia, loss of habitat means that natural processes such as fire have become a disproportionately larger threat to fauna [1]. The Australian fauna have adapted to the destructive effects of fire in part by seeking refuge in surrounding habitat [2]. However, the destruction and fragmentation of habitat by humans has resulted in less available refugia, especially for species with small ranges [3, 4]. Climate change is predicted to increase the frequency and intensity of wildfires, further exacerbating the risk [5]. With this comes an additional threat, especially for small and medium-sized mammals: increased predation by invasive carnivores [6]. Feral cats in particular can travel large distances specifically to hunt within a recently burned area [7].

With habitat loss accelerating and the frequency and intensity of fires increasing, it is critical that conservation measures for threatened species in fire-prone areas are improved [8, 9]. It is important that the specific needs of threatened species are understood, thereby allowing existing conservation areas to be managed in such a way as to reduce the likelihood of their extinction. Species with small ranges tend to be rare within these ranges, increasing the risk of extinction [10]. Given that these species are likely highly habitat-specific, investigating their fine-scale habitat use may be beneficial for conservation management. Additionally, while the effects of fire on both vegetation (e.g., [11, 12]) and small mammals (e.g., [13, 14]) in Australia have been extensively studied, relating the two may be useful for best conservation practice. Of particular concern are threatened wildlife species existing in restricted, fire-prone habitats.

The silver-headed antechinus (*Antechinus argentu*s) is one such species. When described in 2013, it was known from a small area in the eastern part of Kroombit Tops National Park in central eastern Queensland, Australia [15]. Despite extensive trapping at Kroombit and some other montane areas of similar habitat in central Queensland since its discovery, only two populations of the species are known: one at the type location, an ~10 km^2^ area within Kroombit Tops National Park, central eastern Queensland, Australia; and one at Blackdown Tableland National Park, ~200 km to the west of Kroombit [16]. As well as having a highly restricted range, concern for the species’ status is heightened by males being semelparous—all die at the end of a highly synchronised annual breeding period in late June-early July of each year, eliminating any chance of breeding again and effectively halving the population every year [16, 17]. In addition, other threatening processes are known from its habitat including feral predators and grazing from cattle and horses. Due to these factors, *A*. *argentus* was listed as Vulnerable under the Queensland *Nature Conservation Act 1992* in 2015.

The present study was undertaken on the type population of *A*. *argentus* at Kroombit Tops. Just months after *A*. *argentus* was described, the majority of its habitat at Kroombit Tops was burnt by a wildfire. Research on other *Antechinus* species indicates that a lengthy period of vegetation regrowth is needed for populations to recover after a fire (see [18, 19, 20]), and a recent study on *A*. *argentus* hypothesised that this species may be sensitive to fire [16]. Particularly concerning is that although Kroombit Tops comprises a 74.6 km^2^ area, and in spite of an ongoing, extensive trapping effort of ~10,000 trap nights by us and others, *A*. *argentus* is only known from a very small (<10 km^2^) section of the national park even though similar habitat is much more extensive. We therefore hypothesised that *A*. *argentus* may have specific plant-related habitat preferences.

The present study related detailed post-fire plant community composition and structural data to the distribution and abundance of one of only two known populations of *A*. *argentus*, within its highly restricted range at Kroombit Tops National Park in Queensland, Australia. We aimed to investigate whether:

1. Differences in plant community composition and structure following a fire correlate with differences in capture rates of *A*. *argentus*;
2. Plant community composition and structure influence the occurrence of *A*. *argentus*;
3. *A*. *argentus* abundance changed as the post-fire habitat recovered.

By relating detailed vegetation data to our small mammal trapping data, we aimed to uncover abundance and habitat preference patterns of *A*. *argentus* within its small range at Kroombit Tops. We compared our research to existing studies that took similar approaches, and discuss whether this approach may be useful for effective management of threatened wildlife with small, isolated populations in fire-prone habitats.

## Materials and methods

### Study sites

Kroombit Tops National Park is a montane plateau 70 km SSW of the city of Gladstone in Queensland, Australia. Although it is situated in the subtropics, Kroombit Tops has been described as a “mesic temperate island” [21, 22]. Mean annual precipitation at the *A*. *argentus* type locality is estimated at 1400–1800 mm [15], and is mostly concentrated to the summer months, when temperatures are warm to hot. Winters are fine and cool, and frosts are relatively common [15].

**Fig 1:**
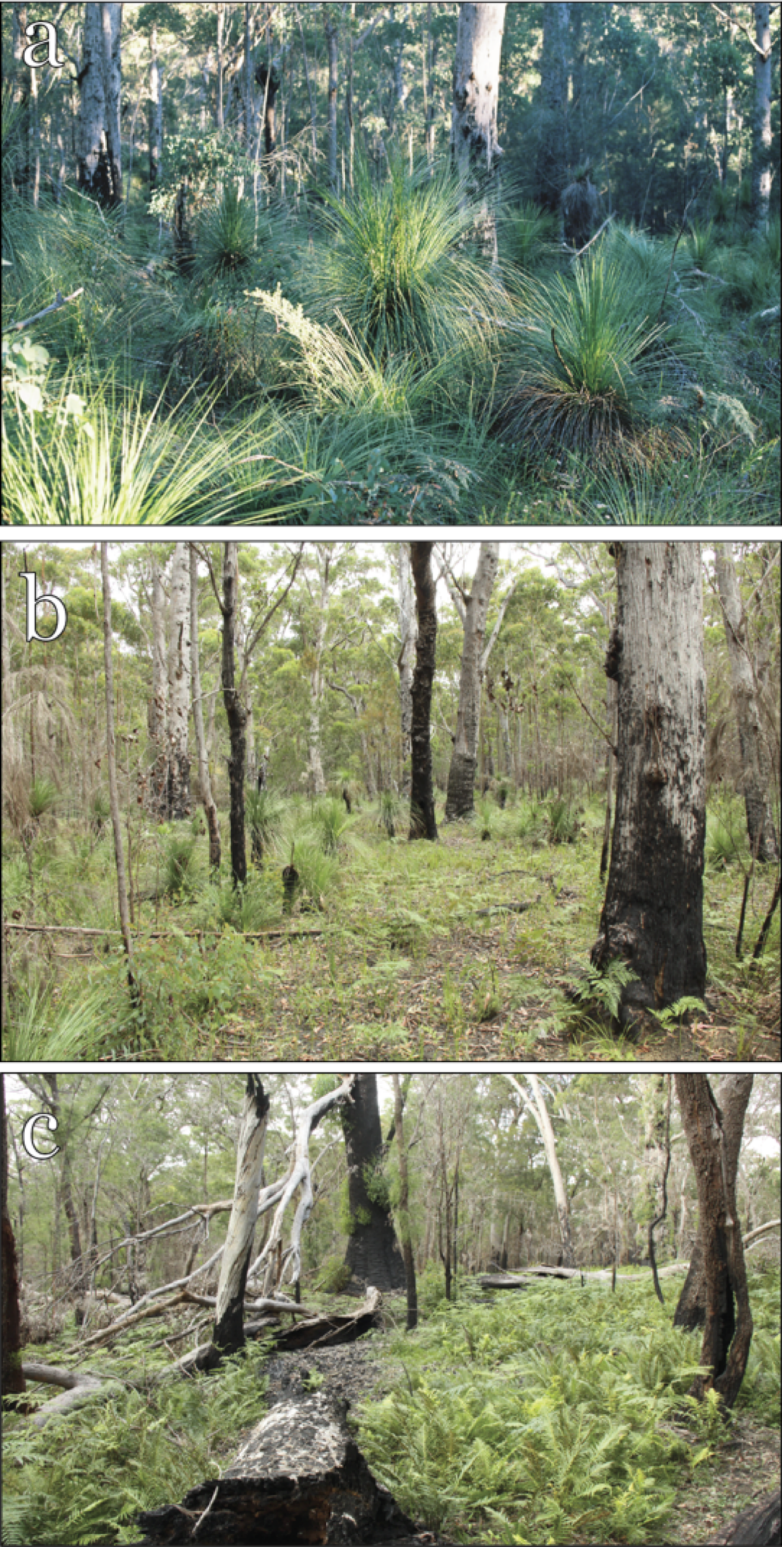
(a) The Lookout Unburnt site, (b) Lookout Burnt site, and (c) Northern site at Kroombit Tops National Park. Photographic credit to Eugene Mason and Harry Hines.

The present study was undertaken at two sites ~6.5 km apart that lie on the eastern edge of a gently undulating sandstone plateau at an elevation of 850–900 m which is bounded on the eastern side by an escarpment with cliffs up to 50 m in height [15]. An ongoing, extensive trapping effort of ~10,000 trap nights has been undertaken by us and others, yet *A*. *argentus* at Kroombit Tops is only known from these two small sites. The habitat at the two sites and the intervening area is broadly described as *Eucalyptus montivaga* and *Corymbia trachyphloia* forest with a grassy, ferny or shrubby understory [15] (Regional Ecosystem 12.9–10.20, Queensland Herbarium [23]). In October 2013, both sites were burnt by a wildfire. The entire Northern site (lat. 24.355 S, long. 151.005 E) was burnt with moderate to high intensity (as defined for forest ecosystems in southeast Queensland by Queensland Parks and Wildlife Service [QPWS] 2013, p16), while only half of the southern or Lookout site (lat. 24.396 S, long. 151.044 E) was burnt, with mostly moderate intensity. Because half of the Lookout site remained unburnt due to an intervening dirt road (Kroombit Forest Drive) that acted as a fire break, we elected to subdivide the site (into halves) along this road for the present study so that it comprised two smaller sites: the Lookout Burnt and Lookout Unburnt sites. Prior to the October 2013 wildfire, the Northern and Lookout Burnt sites were last burnt in a wildfire in September 2001, and a planned burn in March 2008 (QPWS fire mapping), although no information is available on intensity at our study sites. However the Lookout Burnt site was either unburnt during the 2008 planned burn or burnt at a very low severity, based on the size and presence of fire-sensitive trees and shrubs at that site in June 2013 (assessed from a series of HBH photographs). The Lookout Unburnt site falls within a fire management block attributed with being burnt in a planned burn in 2011 (QPWS fire mapping), but visual assessments (degree of skirting on grasstrees, persistence of fire-scarring on trunks of *E*. *montivaga–*see for example Fig 1) strongly suggest that the area falling within our trap grid was not burnt at that time. There are no other records of fires in that block as far back as 1991, indicating that the Lookout Unburnt site is long-unburnt (20+ years).

### Vegetation surveys

Vegetation surveys were undertaken twice (November 2014 and January 2016) to capture the change in vegetation over time following the fire. The layout of the surveys followed already-established mammal trapping grids at the three sites, as per Mason et al. [16]. Six transects formed the Northern site, and three transects each formed the Lookout Burnt and Lookout Unburnt sites. On each of these transects, five 10 m^2^ vegetation plots were laid out at equally spaced intervals (see Trapping surveys section below for spatial arrangement) as per Peet et al. [24], totalling 30 plots at the Northern site, 15 plots at the Lookout Burnt site, and 15 plots at the Lookout Unburnt site. Within each of these vegetation plots, a count of all tree and shrub species was undertaken and the diameter at breast height (DBH) of all trees and shrubs >10 cm was measured (DBHs <10 cm were recorded as 10 cm) from which tree basal area was derived.

Ground cover of eight variables (shrubs, grasses, ferns, herbs, bryophytes, litter, charcoal, bare soil) was estimated as the percentage of each of three randomly placed 1 m^2^ quadrats inside each 10 m^2^ plot. The percentage of ground concealed by the aboveground projection of each variable was estimated [24]. No single ground cover variable could exceed 100%, but total ground cover estimates of all variables within a quadrat could exceed 100% [25].

### Trapping surveys

Small mammal trapping was undertaken at the three sites for a week of each month from March to September in 2014 and 2015. We used aluminium folding traps (Type A Elliott traps, 23 x 9 x 8 cm, Elliott Scientific, Upwey, Victoria, Australia), and baited them with a standard mixture of oats, peanut butter and vegetable oil [26]. The trapping grid consisted of 150 traps laid out along the six transects at the Northern site, 75 along the three transects at the Lookout Burnt site, and 75 along the three transects at the Lookout Unburnt site. The layout of traps was such that every fifth trap location formed the centre of one of the 10 m^2^ vegetation plots. Traps were opened for three nights of each monthly trapping survey period. This totalled 450 trap nights at the Northern site each month, and 225 trap nights at each of the Lookout Burnt and Lookout Unburnt sites for each month. If any *A*. *argentus* individuals were captured, traps were closed for the following night to reduce our disturbance of the population, and to allow the animals to forage naturally.

### Data analyses

Because *A*. *argentus* is only known at Kroombit Tops from two areas (and one of these with adjacent burnt and unburnt sites), the scope of our data analyses was unavoidably constrained–an issue for most threatened species, but particularly an issue for species like *A*. *argentus*, which have only recently been discovered and are already threatened. Furthermore, highly limited *A*. *argentus* captures in 2015 (16 captures of five individuals [two females and three males], [16]) precluded formal comparative vegetation and *A*. *argentus* capture analysis of the 2015 data. These limitations notwithstanding, comparative statistical analyses were undertaken using the November 2014 vegetation data and the March-September 2014 trapping data.

For the plant species composition data, we first square root transformed the data to reduce the dominant contribution of abundant species. We then constructed a resemblance matrix using the Bray-Curtis similarity statistic [27]. Utilising the permutational multivariate analysis of variance (PERMANOVA) pair-wise test function in Primer 7 (version 7.0.1 with add-on PERMANOVA+1), we tested for differences in plant species composition between the three sites [28–30]. We treated the 10 m^2^ vegetation plots as nested within transects, and transects nested within sites. To visualise the differences between vegetation plots, sites and *A*. *argentus* captures, we generated a non-metric multidimensional scaling (nMDS) bubble plot, with each data point representing one of the 10 m^2^ vegetation plots, and the size of the bubbles representing the number of *A*. *argentus* captures in 2014. As the vegetation plots were located at every fifth trap location, we counted the *A*. *argentus* captures from the trap in the centre of the plot as well as two traps either side of the plot in the transect, following our assumption that these individuals would be accessing proximate areas as habitat.

For vegetation structure (ground cover [%] and tree basal area [m^2^]) data, we constructed a resemblance matrix using the rank transform and the Euclidean distance similarity measure to avoid strong skewness in the distribution over samples, and again tested for differences between the three sites using the PERMANOVA pair-wise test function [28–30]. To visualise differences in the structure data between vegetation plots, sites and *A*. *argentus* captures, we generated a principal component analysis (PCA) bubble plot, again representing each 10 m^2^ vegetation plot as a data point and number of *A*. *argentus* captures (as derived above) determining the size of bubbles.

To assess the relative importance of specific predictor variables for the capture (and therefore occurrence) of *A*. *argentus* at our sites, we developed two boosted regression tree (BRT) models [31, 32]. BRT models are simple classification or rule-based models that use a series of binary splits dependent on predictor variables to partition observations into groups based on similar values in the response variable [33]. The boosting algorithm then iteratively fits models to the data in a forward, stage-wise procedure. This method allows for different types of predictor variables to be included in a single model. For our BRT models, we represented the response variable of *A*. *argentus* captures as presence or absence data, and therefore used a Bernoulli distribution.

The first model included the species abundance data, and the relative influence of each species is shown in Table 1. The second boosted regression model included the ground cover (%) and tree basal area (m^2^) data, as well as the average Bray-Curtis similarity for each 10 m^2^ vegetation plot [27]. To obtain this value, the Bray-Curtis similarity of ground cover and tree basal area of each vegetation plot was compared to every other vegetation plot in the survey, then the average of these values was calculated for each vegetation plot. The relative influence of each of these variables is shown in Table 2. We also show the number of trees needed in each of the BRT models and the estimates of the correlation between observed and expected response variables for both of the final models. The BRT models were fitted in the R statistical computing program version 3.2.2 using the gbm package version 2.1.1 [33, 34].

**Table 1:** Boosted regression tree model. Summary of the relative contributions (%) of predictor variables (tree and shrub species abundance) developed with cross validation on data using 1400 trees, tree complexity of 2 and learning rate of 0.004.

Variable	Relative influence (%)
*Xanthorrhoea johnsonii*	38.3
*Corymbia trachyphloia*	27.4
*Allocasuarina torulosa*	15.5
*Elaeocarpus reticulatus*	11.2
*Eucalyptus montivaga*	7.6

**Table 2:** Boosted regression tree model. Summary of the relative contributions (%) of predictor variables (average Bray-Curtis values, tree basal area and ground cover [%]) developed with cross validation on data using 1200 trees, tree complexity of 2 and learning rate of 0.004.

Variable	Relative influence (%)
litter	41.2
Average Bray-Curtis	14.2
herbs	14.1
grasses	14.0
tree basal area	6.4
ferns	4.7
charcoal	3.6
bare soil	1.3
dead wood	0.6

## Results

### 1. Do differences in plant community composition and structure following a fire correlate with differences in capture rates of *A*. *argentus*?

**Fig 2:**
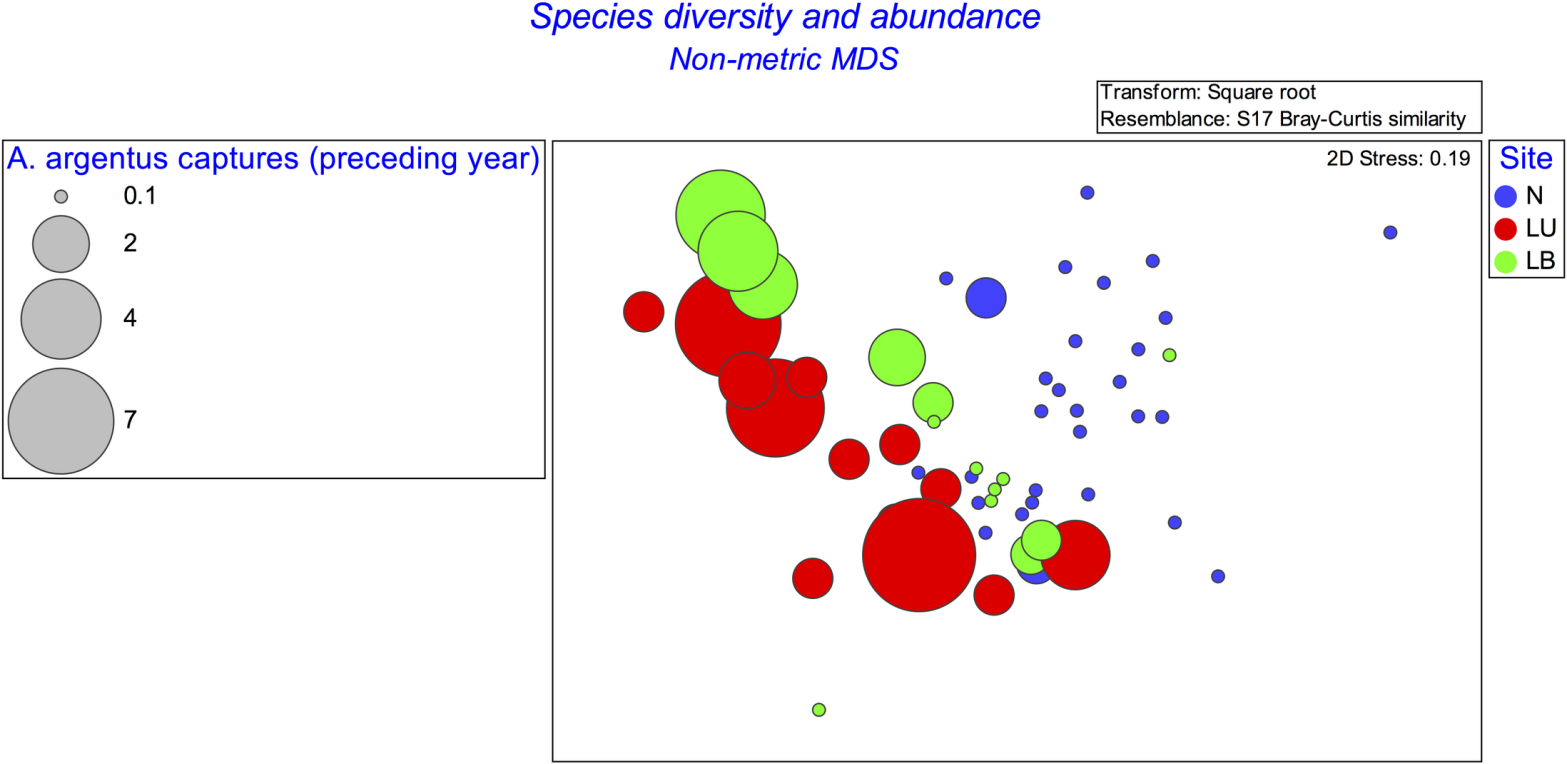
Non-metric multidimensional scaling (nMDS) bubble plot of vegetation species diversity at each 10 m^2^ vegetation plot. The size of the bubbles indicates the number of *A*. *argentus* captures during 2014. N = Northern, LU = Lookout Unburnt, LB = Lookout Burnt. 0.1 represents vegetation plots with nil captures.

While the three sites were not unambiguously separated based on nMDS of vegetation species diversity, two-dimensional structure was suggested by the grouping of the Lookout Unburnt (red) and Lookout Burnt (green) vegetation plots into diagonal lines (Fig 2). Additionally, there was structure in the number of *A*. *argentus* captures, as indicated by the size of the bubbles representing each vegetation plot. In particular, the majority of captures were made at the Lookout Unburnt site, followed by the Lookout Burnt site. Multivariate analysis of species abundance showed a significant difference between the Northern and Lookout Unburnt sites in 2014 (PERMANOVA, t = 2.05, *P* = 0.01), while evidence against the null hypothesis was weak for comparison of the Northern and Lookout Burnt sites (PERMANOVA, t = 1.52, *P* = 0.08), and weaker still for comparison of the Lookout Burnt and Lookout Unburnt sites (PERMANOVA, t = 1.15, *P* = 0.39).

**Fig 3:**
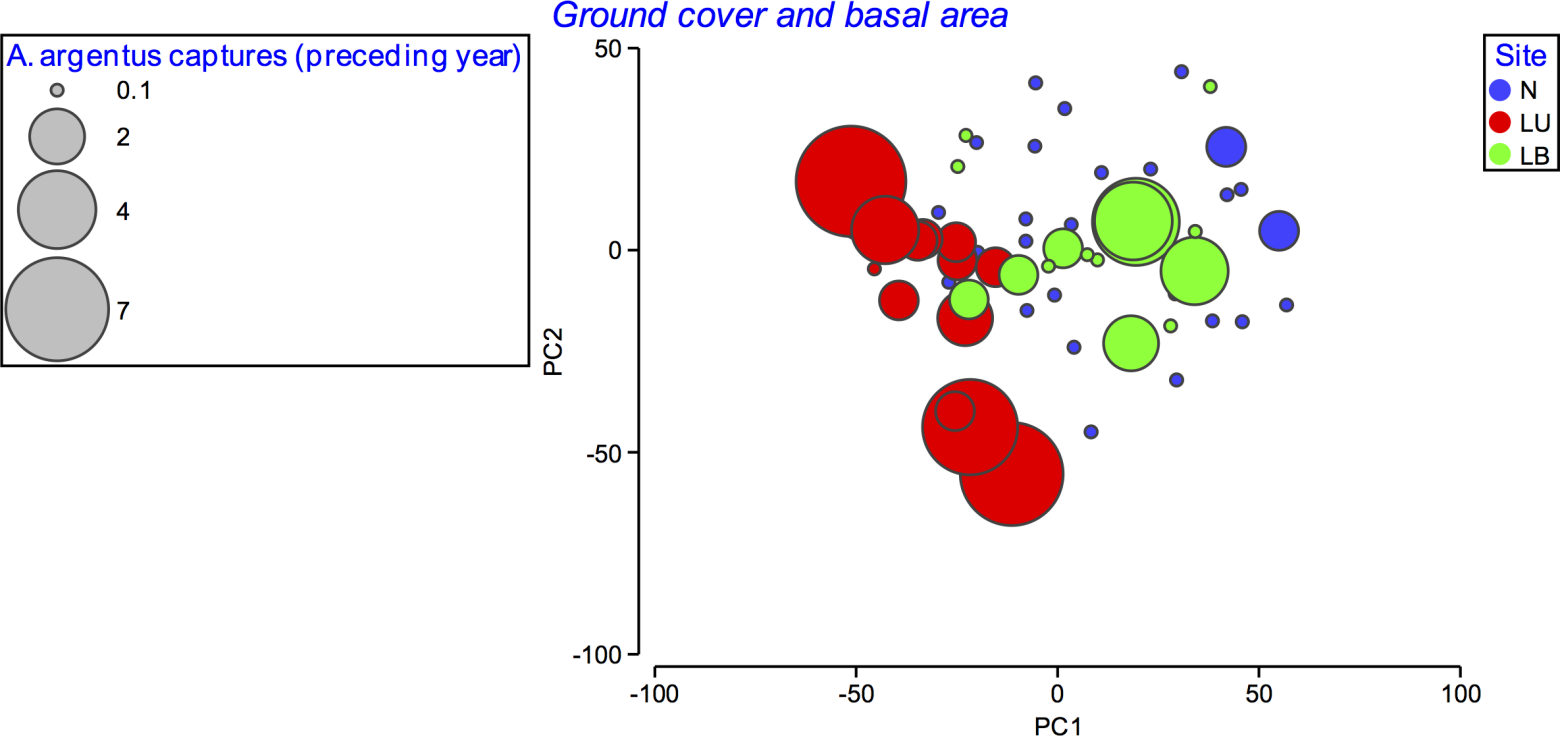
Principal component analysis (PCA) bubble plot of ground cover (%) of litter, grasses, ferns, bare soil, charcoal, bryophytes, herbs, shrubs and dead wood and and tree basal area (m^2^) at each 10 m^2^ vegetation plot. The size of the bubbles indicates the number of *A*. *argentus* captures during 2014. N = Northern, LU = Lookout Unburnt, LB = Lookout Burnt. 0.1 represents vegetation plots with nil captures.

Similar results were found for the vegetation structure (ground cover and tree basal area). Again, explicit separation was not evident between the three sites based on PCA, although the Lookout Unburnt (red) vegetation plots were grouped in a diagonal line (Fig 3). Multivariate analysis of vegetation structure showed there was a significant difference between the Northern and Lookout Unburnt sites in 2014 (PERMANOVA, t = 2.92, *P* = 0.01), but evidence against the null hypothesis was weak for comparison of the Lookout Burnt and Lookout Unburnt sites (PERMANOVA, t = 2.83, *P* = 0.09) and weaker still for comparison of the Northern and Lookout Burnt sites (PERMANOVA, t = 1.12, *P* = 0.34).

### 2. Does plant community composition and structure influence the occurrence of *A*. *argentus*?

Nineteen tree and shrub species were recorded from the vegetation plots, but of these only five were found to exhibit a relative contribution to *A*. *argentus* captures in the tree and shrub diversity and abundance BRT model (Table 1). The model indicated that the most important tree or shrub species for the capture of *A*. *argentus* was the grasstree *X*. *johnsonii* (38.3%, Table 1) followed by the bloodwood *C*. *trachyphloia* (27.4%, Table 1). The estimate of the correlation between observed and predicted response variables was 74%.

The partial responses of *A*. *argentus* captures for the six most influential variables in the ground cover (%), tree basal area and average Bray-Curtis model indicated a species that favours areas with ground cover dominated by high amounts of leaf litter and the presence of herbs and grasses, and that are relatively different to the overall habitat (as indicated by lower average Bray-Curtis similarity values). By far the most important variable was leaf litter (41.2%, Table 2). The estimate of the correlation between observed and predicted response variables was 87%.

### 3. Does *A*. *argentus* abundance change as the post-fire habitat recovers?

**Fig 4:**
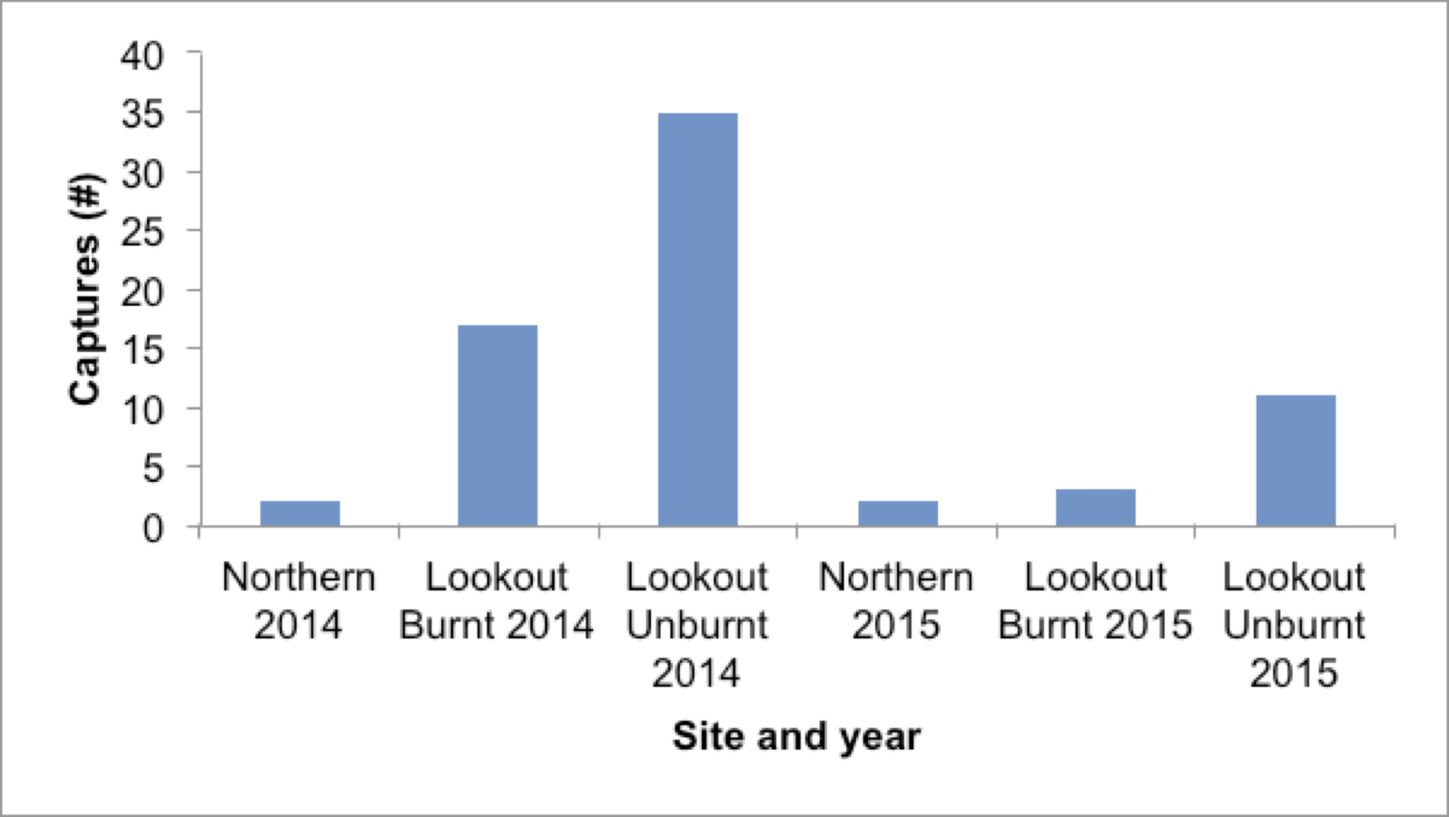
*A*. *argentus* captures at Kroombit Tops in 2014 and 2015.

Despite a longer period of post-fire vegetation recovery, *A*. *argentus* captures dropped dramatically overall from 2014 (54 captures of 15 individuals) to 2015 (16 captures of 5 individuals) (Fig 4). The highest capture rate per site for both years was at the Lookout Unburnt site (35 captures of 9 individuals in 2014; 11 captures of 3 individuals in 2015). Across both years, more captures were recorded at the Lookout Burnt site (2014: 17 captures of 6 individuals; 2015: 3 captures of 3 individuals) than the Northern site (2014: 2 captures of 2 individuals; 2015: 2 captures of 1 individual).

Average ground cover (%) of the most influential structure variable for *A*. *argentus* captures in 2014 (leaf litter) increased at the Northern (55.9–87.3%) and Lookout Burnt (64.2–82.1%) sites between vegetation surveys, but decreased slightly at the Lookout Unburnt site (98.4–87.6%) (Table 3).

**Table 3:** Average leaf litter cover.

Date	Northern	Lookout Burnt	Lookout Unburnt
Nov 2014	56.0	64.2	98.4
Jan 2016	87.2	82.1	87.5

## Discussion

Differences in plant community composition and structure between recently burnt and unburnt habitat were found to correlate with the occurrence of *A*. *argentus*. *A*. *argentus* appears to have a preference for habitat not recently burnt. Within unburnt habitat, the fine-scale occurrence of the species was influenced by ground cover attributes and the presence and abundance of certain plant species, in particular the grasstree *X*. *johnsonii*. The data presented here may prove important for habitat management of a highly restricted, isolated population of this threatened species. Our results also highlight the potential utility of relating detailed vegetation data to the ecology of threatened species that have small ranges within fire-prone areas. These aspects of the data are addressed in more detail below.

Plant community composition and structure differed between the three sites, with the strongest differences between the Lookout Unburnt site and the Northern site (Figs 2 and 3). The Northern site was completely burnt at moderate to high severity in October 2013. This pattern was paralleled by the 2014 *A*. *argentus* capture rate data, which was highest at the Lookout Unburnt site, and lowest at the Northern site (Figs 2 and 3). The three sites are proximate and well connected with similar habitat; plausibly, these differences could largely be explained by the effects of the October 2013 wildfire (five months prior to the commencement of the present study) on habitat variables, in turn influencing *A*. *argentus* capture rate. The effects of fire on other *Antechinus* species are relatively well-documented (e.g., [18, 20, 35–39]). However, these studies focused on *Antechinus* species with comparatively larger geographic ranges than *A*. *argentus*, the latter only being known from two isolated montane plateaux. Moreover, the abundance of *A*. *argentus* at Kroombit Tops is apparently very low. A study on *A*. *minimus* found that patchily-burnt areas were recolonised at low abundance after a fire, but areas that were fully burnt resulted in local extinction of the species [20]. Likewise, Fox [36] found that a population of *A*. *stuartii* recolonized an area six months after a fire, but at a lower abundance (57%) than the pre-fire population, and proposed that this might be a response to changes in the vegetation structure and composition due to fire.

In temperate and semi-arid areas of Australia, studies have found that plant community composition and structure influences small mammal distribution [40–42]. A number of studies have noted the importance of dense low-level foliage for *Antechinus* species (e.g., [43, 44–46]), but few have described the preferences of *Antechinus* species for specific vegetation types. Banks et al. [47] found that abundant large eucalypts had a positive effect on *A*. *agilis* population size, with populations decreasing as the distance from eucalypt-dominated forest increased and transitioned into other forest types. Studies on *A*. *stuartii* have suggested this species also has a preference for habitat containing grasstree (*Xanthorrhoea*) species [48–50]. Likewise, Swinburn et al. [19] found that *A*. *flavipes* use *Xanthorrhoea* as important foraging and nesting resources, and Whelan et al. [51] found evidence of frequent visits from *A*. *stuartii* to flowering inflorescence stems of *Xanthorrhoea*. Furthermore, in post-fire environments these species showed a preference for habitats with numerous surviving *Xanthorrhoea* individuals. Like several of its congeners and the very closely related *A*. *flavipes*, our study found that the grasstree *X*. *johnsonii* was the most important floristic predictor for captures of *A*. *argentus*. Grasstrees provide habitat for at least 315 invertebrate species [52]. While other *Antechinus* species have diverse insectivorous diets, sometimes supplemented with soft, ground-dwelling invertebrates or small vertebrates [53–55], *A*. *argentus* has a diet dominated by just two invertebrate orders: Coleoptera and Blattodea [56]. Both of these are typically found in unburnt or regenerating *Xanthorrhoea* with dense skirts [19, 37], indicating that *X*. *johnsonii* may provide an important foraging resource for *A*. *argentus*. While we were not able to directly observe any *A*. *argentus* foraging or nesting in an *X*. *johnsonii*, we did observe an *A*. *argentus* individual launching outward at human head height from within a large *X*. *johnsonii* skirt at the Lookout Unburnt site during a trapping survey (pers. obs.).

In contrast to other species of *Antechinus*, *A*. *argentus* capture rates dropped drastically from 2014 to 2015 (Fig 4) despite a longer time period since fire. Leaf litter markedly increased at both of the burnt sites by 2016 (Northern and Lookout Burnt) (Table 3). We found that leaf litter was the most important ground cover variable in terms of relative contribution for the occurrence of *A*. *argentus* at Kroombit Tops (Table 1). A thick leaf litter layer has been shown to influence occurrence of *A*. *flavipes* [57]. Arthropods found in leaf litter are a necessary food component for carnivorous small mammals including *Antechinus*, and therefore this might be partially explained by the role of leaf litter for the abundance of invertebrate prey [58, 59]. The lower amount of leaf litter at the burnt sites in November 2014 is likely explained by its removal through combustion in the October 2013 wildfire. The volume of flammable leaf litter in the ground cover assemblage can be a determining factor in the initiation of wildfires, and leaf litter accumulation has been shown to be a function of the regeneration age of eucalypt forests since a fire [60, 61]. This is reflected in the increase in leaf litter ground cover at the two burnt sites from Nov 2014 to Jan 2016. However, the *A*. *argentus* population did not become more abundant in the burnt sites as the time since fire increased. In fact, it apparently declined across all three sites from 2014 to 2015 (Fig 3). Plausibly, this could indicate some broader effects of recent fire damage, such as increased predation by introduced fauna.

Studies have found evidence that the loss of vegetation cover caused by higher intensity fire allows for a higher proportion of the habitat to be available to feral predators [7]. In particular, feral cats (*Felis catus*) have been shown to travel large distances to reach recently burnt habitat, amplifying predation on exposed native species in the absence of sufficient cover [6, 62, 63]. Recent evidence has linked the declines of small mammal species with these amplified feral predation effects due to fire, as well as the effects of grazing by introduced herbivores [64]. During the present study, we directly or indirectly observed cattle (*Bos taurus*), pigs (*Sus scrofa*), horses (*Equus caballus*), cats (*Felis catus*) and dogs/dingos (*Canis* sp.) at the study sites. Grazing by cattle and horses appeared to considerably reduce or slow recovery of palatable species post-fire (mostly grasses), resulting in open areas with very cropped grass between patches of fern, or shrub or fallen timber cover (pers. obs.). Cats are widespread at Kroombit Tops (QPWS unpublished data) and limited camera trapping at the Lookout sites in late 2015 confirmed the presence of one large individual. The combined pressures of grazing and predation from introduced carnivores in a post-fire landscape may be explanatory factors in the decline of the Kroombit *A*. *argentus* population, but this is a preliminary hypothesis until future work investigates direct interactions.

While the approach of the present study emphasised vegetation composition, we suggest that future research focus on structural habitat elements such as the size and shape of vegetation, for example the diameter and leaf density of grasstree crowns. We also suggest that relating invertebrate species diversity in grasstree crowns to *A*. *argentus* prey preference may reveal important patterns. Nevertheless, the apparent importance of specific habitat components such as *X*. *johnsonii* for the occurrence of *A*. *argentus* highlights the utility of relating fine-scale habitat data to wildlife occurrence. This data will allow future trapping surveys to be targeted in areas of similar habitat in the hope of uncovering additional populations of the species. It also adds to a growing body of literature concerning the response of wildlife to changing fire regimes. Recent work has suggested that fire is likely a prominent feature in the evolutionary history of *Antechinus*, finding that smoke and a substrate of ash and charcoal may act as a cue for the onset of torpor in *A*. *flavipes* [65]. However, in contrast to congeners (see [18, 20, 36]), our results indicate that long-unburnt vegetation is important for *A*. *argentus*. The present study therefore highlights the importance of a localised approach to fire management, especially in habitats that house threatened species. The future of *A*. *argentus* at Kroombit Tops is uncertain; a trapping survey in June 2016 at our three study sites resulted in just one *A*. *argentus* capture (data not shown). As one of only two known populations of the species, effective conservation management is vital if it is to survive. As global habitat loss accelerates, unfortunately there are likely to be more species facing comparable threats to *A*. *argentus*.

The interactions between fire and biodiversity are complex [66, 67]. Many plants and animals require fire to survive, but even in fire-prone habitats some species are highly sensitive to fire [68]. A landmark study by Martin and Sapsis [69] hypothesised that temporal and spatial variation in fires can promote biodiversity by creating a wider variety of ecological niches available for species. A growing body of literature has supported this hypothesis (see [67, 70, 71]). However, pyrodiversity doesn’t always promote biodiversity. A recent study found that in Australian semiarid eucalypt woodland, increasing variation in fire regimes didn’t correlate with increasing bird diversity because long-unburnt vegetation provided disproportionately important habitat [72]. Nevertheless, the landscape of Australia has changed dramatically over the last 250 years as a result of human activity, and as a result the ability for fauna to sustain the effects of fire has likely been lowered [73]. As an important driver of ecology in Australia, appropriate fire regimes will benefit from research into the critical limits of severity and patch size [68]. This is a relatively new area of research, but studies suggest that fire management approaches are best tailored to local conditions (see [74, 75]), and will rely on specific studies [68] such as the present one. Understanding the effects of fire on threatened species is vital if we are to properly ensure their survival, as ongoing global climate change may ensure increased fire frequency and intensity [5]. This is likely to be especially important for species that are rare, highly restricted and that inhabit fire-prone areas. For these species, data quality (i.e., spatial resolution) appears to prevail over data quantity (i.e., number of data) [76], and therefore a more fine-scale approach to habitat mapping may inform conservation management strategies. Additionally, fine-scale habitat data can be utilised to predict unsurveyed sites of high potential of occurrence [77]. Effective management of conservation areas that house rare and endangered species is becoming increasingly important, and a greater understanding of their distribution and localised habitat use will help to mitigate threats such as invasive predators and fire.
